# Comparative Assessment of Soil Contamination by Lead and Heavy Metals in Riparian and Agricultural Areas (Southern Québec, Canada)

**DOI:** 10.3390/ijerph7083100

**Published:** 2010-08-05

**Authors:** Diane Saint-Laurent, Marlies Hähni, Julien St-Laurent, Francis Baril

**Affiliations:** 1 Géographie et Laboratoire de recherche en géomorphologie fluviale et sols, UQTR, 3351, boul. des Forges, C.P. 500, Trois-Rivières, QCG9A 5H7, Canada; 2 UQTR, Département de Chimie et Biologie, Sciences de l’Environnement, C.P. 500, Trois-Rivières, QC G9A 5H7, Canada; E-Mails: Marlies.hähni@uqtr.ca (M.H.); francis.baril@uqtr.ca(F.B.); 3 Environnement et Développement durable, Ville de Trois-Rivières, C.P. 368, Trois-Rivières, QC G9A 5H3, Canada; E-Mail: julien.st-laurent@V3r.net

**Keywords:** contamined soils, riparian and agricultural zones, abandoned mines, hydrocarbons-layer, lead and heavy metals, wildlife and human health

## Abstract

Soils contaminated with hydrocarbons (C_10_–C_50_), PAHS, lead and other heavy metals were recently found in the banks of two major rivers in southern Québec. Alluvial soils are contaminated over a distance of 100 kilometers. Eight sampling sites, including some located in agriculture areas (farm woodlots) have been selected to compare air pollution (aerosol fallout and rainout) and river pollution values. The concentrations detected in soil profiles for As, Cd and Pb vary between 3.01 to 37.88 mg kg^−1^ (As), 0.11 to 0.81 mg kg^−1^ (Cd) 12.32 to 149.13 mg kg^−1^ (Pb). These metallic elements are considered highly toxic and can harm wildlife and human health at high levels. The maximum concentration of Pb (149.13 mg kg^−1^) in soils of the riparian zone is twelve times higher than the average Pb concentration found in a natural state evaluated at 15.3 mg kg^−1^ (SD 17.5). Pb concentrations in soils of agricultural areas (woodland control sites) range between 12 and 22 mg kg^−1^, and given that these values are recorded in surrounding cultivated land, the issue of the quality of agricultural products (crops and forage) to feed livestock or destined for human consumption must be further addressed in detail.

## 1. Introduction

Mining operations and industrial activities are causing serious damage to terrestrial and aquatic environments and are a main source of environmental contamination. In many cases, these activities have led to the major pollution and contamination problems found today [1–3]. Organic substances (e.g., PAHs, PCBs, hydrocarbons or petroleum products) and heavy metals can be present for extended periods in soils, groundwater, lakes or streambed sediments [4–6]. Also, these pollutants in soils and sediments have a direct impact on aquatic systems and water quality, particularly in areas subject to severe contamination. These contaminants in high concentrations are considered to affect wildlife, causing eggshell thinning, tumors and other deformities [7]. Elevated concentrations of pollutants in aquatic environments and soils are known to endanger ecosystems and introduce a potential risk to human health [8]. Lead and some heavy metals (e.g., As, Cd, Cu, Zn) in high concentrations have an adverse effect on human health, causing profound biochemical changes in the body and affecting the central nervous system [9–11]. The study by Tripathi *et al.* [10], for example, shows a strong correlation between the decreasing trend in the hemoglobin content with increasing blood Pb concentrations in children (3–6 years), which can potentially affect the metabolism and cognitive development of children.

**Figure 1.**
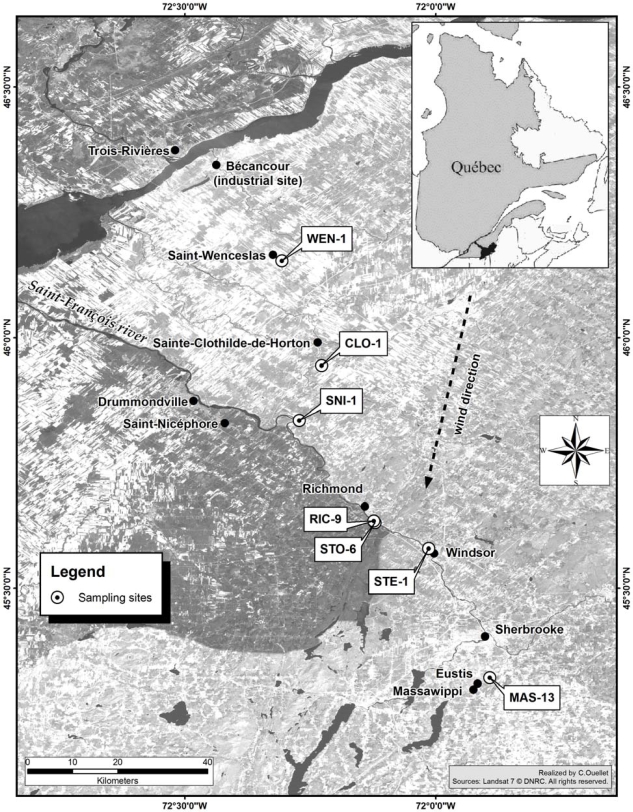
Location of sampling sites of alluvial soils (riparian area) and soils in agricultural areas (woodlots). Some sites are located in the St. Lawrence Lowlands and others in the Appalachian region.

**Figure 2.**
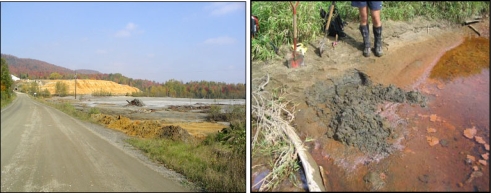
(a) View from the landfill of the former Eustis mine. The site serves as a pulp and paper waste disposal and storage site. (b) High concentrations of residual iron and other tailings on a riverbank of Massawippi River near Eustis mine, Southern Québec (Photos by D. Saint-Laurent, 2005). (a) Former Eustis mine (b) Bank (left) of Massawippi River

This study aims to assess the degree of metal contamination by various heavy metal pollutants in riparian soils along the Saint-François and Massawippi Rivers (Figure 1), including three areas surrounding agricultural sites (farm woodlots). Riverbank contamination covers several dozen kilometers along the Saint-François and Massawippi Rivers, and some pollutants it also found in cultivated land (e.g., crop production) in the banks. The Richmond, Windsor and Eustis area soils emerge as the most contaminated sites along the riverbanks [12] and are mainly impacted by heavy metals and other pollutants (e.g., hydrocarbons, Pb, PAHs). For instance, the contamination by hydrocarbon substances (C_10_–C_50_) extending over more than 100 kilometers of riverbanks between the municipalities of Eustis and Drummondville [12]. The hydrocarbon contamination takes the form of horizontal layers in the banks with variable thicknesses (between 2–3 cm and over one meter), and which is easily detectable by its dark color and pronounced oil smell. The main source of contamination appeared to be the former Eustis mine site that closed in 1939 [13]; it is assumed that one or more spills (hydrocarbons and other pollutants) were discharged directly into the river from this site, left unattended for several years. The Eustis site also serves as a pulp and paper waste disposal and storage site (Figure 2). In 2007 and 2008, the site of the former Eustis mine was restored by the Ministère du Développement durable, de l’Environnement et des Parcs du Québec (MDDEP) [14]. The restoration work involved capping mine waste and tailing deposits with an impermeable barrier to reduce runoff and seepage from acid mine tailings.

The main objectives of the study are (1) to evaluate the contamination of alluvial soils and complete the fieldworks that was begun in 2002; and (2) to determine and compare the concentrations of heavy metals in the soils found in riparian areas as well as in agricultural soils in order to assess their respective levels of contamination. The study focused on the total concentrations of heavy metals in soils (As, Cd, Cr, Cu, Ni, Pb, Zn), including some physical and chemical properties of the soil samples (pH, total organic carbon (%), grain size analysis). The study also aimed to supplement data on high levels of Al and Pb that were measured in some alluvial soils and tree-rings of riparian trees [15,16]. Atmospheric fallout is suspected [17], in particular Al dust that may originate from industrial plants in the Bécancour area located some 40–60 km from the study areas. The soil samples taken from the wooded areas in the agricultural zone may potentially be contaminated by surrounding atmospheric pollutants. In this respect, high concentrations of Al and heavy metals (e.g., Pb, Zn) may present a health risk [9,10], therefore it is important to assess the level of contamination of the reference sites (woodlots) in agricultural areas. For this study, the procedure sampling and laboratory analysis follow the criteria and guidelines stipulated by the MDDEP in its Policy on Soil Protection and the Rehabilitation of Contaminated Sites [18], the Centre d’expertise en analyse environnementale of Québec [19], and on Canadian System of Soil Classification criteria [20].

## 2. Industrial and Mining Heritage

The rivers and streams that cross through the industrial, urban and agricultural areas of the Saint-François River basin are affected to varying degrees by the respective emissions. The first water quality control measures determined by the government, in particular for the industrial sector, date back to the 1970s, with other measures being added in 1988 aimed at reducing industrial waste emissions into the air, water and soil [21]. Before 1990, the waters of the Saint-François and Massawippi Rivers were severely contaminated by industrial and agricultural waste. The creation of wastewater treatment plants from 1990 to 2000 [22] in most of the municipalities along the Saint-François River and its main tributaries seems to have helped decrease the concentration levels of several contaminants detected downstream of Drummondville (e.g., PAHs, PCBs, heavy metals, fatty and resin acids, fecal coliform bacteria and turbidity).

Despite these water purification efforts, the Saint-François River is still affected by urban and agricultural pollution [23], and the Massawippi River remains polluted due to agricultural discharges and mine tailings from the former Eustis mine disposal site found along the Massawippi River (Figure 2). Metal contamination and the heavy acidification of the affluents alongside the Eustis mine site have been found [22,24]. The Massawippi River, a major affluent of the Saint-François River, flows near Eustis (the former mining site), which was in operation from 1865 to 1939 [13]. During this period, the ore extracted from the site consisted of copper and chalcopyrite minerals (CuFeS_2_), and from 1889 to 1927, about 34,000 tons of ore were extracted per year [12,13]. Pollution problems appeared in the early years of the mine’s operation. The sulfur emissions from copper ore roasting appear to have affected the surrounding area, with adverse effects on the health of neighbouring communities as well as on cattle, crops, and even trees [13]. Today, more than 70 years after the mine closed, the presence of sulfurous mine waste is the main problem at the Eustis site [25]. The acid mine drainage caused by the oxidation of iron sulfide found in the waste is a major source of contamination of the Eustis creek and of the Massawippi River. The study by Berryman *et al.* [24] also shows high levels of Cd, Cu, Fe, Pb and Zn in the Eustis creek that were respectively 41, 2,490, 67, 25 and 65 times higher than the criteria for protection of aquatic life [26]. The Eustis site was also long left an orphan site and we assume that it has been the site of many illegal discharges, including the hydrocarbon spills now found along the banks of the Massawippi and Drummondville rivers [15].

Though water quality has substantially improved over the last two decades, pollution and degradation problems are still noted in certain areas [18]. For instance, in the Eustis area along the Massawippi River and between Sherbrooke and Richmond, water quality still ranges from satisfactory to below-average along the Saint-François River; at Drummondville, it deteriorates substantially and is considered poor [18]. There are also a number of fish with morphological anomalies, including tumours, lesions and deformations [7,27] resulting from water pollution. Between East Angus and Richmond and downstream of Drummondville, the proportion of fish with anomalies is often well over 5%, a threshold above which fish health is considered precarious [27]. For instance, 20% of the fish downstream of Richmond have morphological anomalies (e.g., tumours), compared to 15% upstream of Drummondville. Obviously, the harvesting of fish with such anomalies should be severely limited for consumption purposes.

## 3. Experimental

### 3.1. Site Description

All the sites in riparian areas are located in the Saint-François River basin, with the exception of the three control sites in the agricultural area. The Saint-François River is the main body of water in this vast catchment (10,230 km^2^), and originates in the Saint-François reservoir lake, which is located in the Appalachian region and flows northward to feed into the St. Lawrence River at Lake Saint-Pierre (Figure 1). The Saint-François and Massawippi Rivers cross through former industrial areas (Sherbrooke, Bromptonville, Windsor) and mines (Eustis, Capelton), which played a key role in the region’s economic development especially during the first half of the 19th century (in particular the mining operations of the Eustis-Capelton-Albert complex) and were considered the cradle of copper mining in Canada from 1856 to 1939 [13]. Today, industrial activity is mainly found around Sherbrooke and Drummondville, while mining activity is limited to a few sites outside the study area. There is also major economic activity centered around agricultural production mainly in the St. Lawrence Lowlands.

### 3.2. Field Sampling

To identify the source of the contamination in alluvial soils (riparian zone) and agricultural areas (woodlots), field work was done between 2002 to 2008 [12,15,16]. For this study, four areas were selected (RIC-9, STO-6, STE-1 and MAS-13) along the riverbanks between the municipalities of Drummondville and Eustis (the Richmond, Windsor and Massawippi sites), and three other areas (WEN-1, CLO-1, SNI-1) located in an agricultural zone (Figure 1). These last three areas follow a southeasterly direction between the Bécancour industrial site and the town of Richmond, at a distance of 30–60 km (Figure 1), and are subject to north and northeasterly winds [28], which likely transport air pollutants over longer distances. The location of all the sites was determined with a Global Positioning System (GPS) and each site was then located on the mapping medium using the ArcGIS software program (versions 8.2 and 9.0).

At the eight sites, 56 soil samples were collected in the riparian zone (Saint-François and Massawippi Rivers) along with 18 soil samples in the agricultural area. For soil profiles (riparian zone), a trench was dug along the riverbank in order to reach the hydrocarbon-contaminated layers, which in some cases were found at a depth of more than one meter. The data collected in the field consisted of the distance separating the trench from the riverbank, the description of the soil profile (e.g., facies and soil texture, color of horizons), and depth of the hydrocarbon-contaminated layers found in the profile. The soil samples were collected at defined depths (e.g., 20, 40, 60, 80, 100 cm) and stored in sterilized bottles and refrigerated containers. They were then sent to independent laboratories (Biolab Inc. and Maxxam Analytic Inc.) for total heavy metals analysis. A portion of the soil samples was used for textural analysis (particle size), soil color (Munsell chart), and chemical properties (pH, total organic carbon) based on the criteria of the Canadian System of Soil Classification and the Canada Soil Information System [20,29].

### 3.3. Physical and Chemical Analysis

At the laboratory, the soil samples were air-dried and passed through a 2-mm stainless steal sieve prior to physical and chemical analysis (particle size, pH, TOC). For grain size analyses, the sandy fraction was obtained by sieving, while the finer fractions were obtained using a hydrometer. The methods used for chemical analyses consisted of determining the pH in CaCl_2_ (0.01M) using a 1:2 soil-solution ratio [30], and the total organic carbon (TOC) content using the method developed by Yeomans and Bremner [31]. For heavy metals analysis, the sampling method consisted of taking samples of soils that were mixed in a receptacle to ensure the homogeneity of the sample. The soil samples were then stored in sterile containers and refrigerated on site. The heavy-metals analyses were done according to procedures approved by Quebec’s Environment Ministry [18]. The samples were dissolved using HNO_3_ and the resulting liquid residue was then analyzed using inductively coupled plasma mass spectrometry (ICP-MS). Blank samples were prepared for every batch of 10 or 12 samples. For heavy metals concentration analysis, the complete procedures included the duplicate analyses required to validate the laboratory tests [19]. A total of 74 samples were used for analysis of heavy metals (As, Al, Cd, Cu, Cr, Ni, Pb, Zn). The analysis protocol was completed by an outside laboratory (Biolab Inc., Maxxam Analytics Inc.) accredited by the MDDEP [18].

## 4. Results and Discussion

### 4.1. Soil Sample Properties and Geochemical Characteristics

Tables 1 and 2 show some of the properties (pH, total organic carbon, grain size) and heavy metal concentrations of the soil samples (*n* = 74) collected in the riparian zone and the agricultural area. Soil pH was relatively acidic with mean and median values of 5.15 and 5.31, respectively, based on the pH logarithmic scale (0–14). The minimum and maximum values obtained were 3.45 and 7.09, respectively (Table 1). Soil organic carbon concentrations are relatively low overall with values range from 0.05 to 1.56% (Table 1). The dominant soil matrix are sandy loam and loamy sand, which yields a fine texture that is a common feature of alluvial soils found along the riverbanks in this area [32,33]. The soil in agricultural areas (woodlots) consists of a sandy matrix (over 90% sand) and soil from reworked eolian deposits [34].

Table 2 shows the concentrations of heavy metals in the soil samples collected at all the sites. Cu, Pb and Zn were found in relatively high concentrations, in some cases even exceeding levels A and B (see Table 3) of the MDDEP’s generic criteria for contaminated land [18]. The high levels of copper in some of the soil samples (e.g., 120 mg kg^−1^) can be easily be explained by the presence of mine tailings from the former copper mines (Eustis-Capelton) found along the Massawippi River (see Table 3). These tailings may have been transported over long distances by the current and deposited along the banks of the Massawippi and Saint-François Rivers [12]. In fact, high concentrations of copper (between 10 ug/L and 75 ug/L) were found in the Massawippi River downstream of the mine sites [25]. Pb and Zn concentrations are also relatively high and may originate from mine tailings and possibly from the polluted inflow from industrial waste located around Sherbrooke or Windsor.

### 4.2. Contamination of Alluvial Soils (Riparian Zone)

The field work along the riverbanks of the Saint-François and Massawippi Rivers reveals that the contamination extends from the former Eustis mine to the municipalities of Richmond and Drummondville (Figure 1). Soils from both riverbanks from Eustis to Drummondville, a distance of 103.5 km, have also been found to be contaminated with hydrocarbons (C_10_–C_50_) [12]; however, no other traces of hydrocarbon contamination were found outside the boundaries of the river sections examinated in the present study (*i.e.*, upstream of the Eustis site and downstream of the Drummondville site). The concentration of heavy metals in alluvial soils varies from one site to the next (Table 3). The samples taken from the Richmond and Ulverton areas (STO-10-6, STO-13-6 and STO-15-6) show the highest levels of contamination (Table 3). The other sites (Windsor and Massawippi) show generally moderate and low metal (e.g., WIN-2-8 and MAS-13-8). According to the MDDEP’s Policy on Soil Protection and the Rehabilitation of Contaminated Lands [18], contaminated soil is categorized based on the following criteria: contamination levels: A–B: residential uses; B–C: industrial uses; >C: use prohibited without treatment. The concentration levels of the different heavy metals analyzed comply with MDDEP standards [18]. For each heavy metal analyzed (As, Cd, Cr, Cu, Ni, Pb and Zn), contamination level B is considered, to be 30 mg kg^−1^ for arsenic, 5 mg kg^−1^ for Cd, 250 mg kg^−1^ for chromium, 100 mg kg^−1^ for Cu and Ni, 500 mg kg^−1^ for Pb and Zn, respectively (see Table 3). These levels create certain usage constraints since MDDEP standards state that any contaminated levels that meet criterion B (B–C level) means that the soils contain inorganic contaminants.

Upstream of Richmond, heavy metals in soil are less concentrated but still present in alluvial soils. The soil samples taken from the Richmond and Ulverton areas (e.g., STO10-6, STO-13-6 and STO15-6) showed relatively high contamination levels, *i.e.*, 120 and 130 mg kg^−1^ for Cu, and 440 and 1,500 mg kg^−1^ for Zn, respectively (Table 3). In the Massawippi area, the values obtained are comparable with those in the Windsor area. Also, the lowest concentration of heavy metals found in some soil samples comes from the Windsor and Richmond areas (WIN-2-8 and RIC-2-8). The total concentration of heavy metals obtained at these sites corresponds to level A criteria (detection limit) and does not represent a potential hazard according to the MDDEP’s contaminated soil criteria [18]. Overall, the results showed low and moderate levels of heavy metals, except for the STO-10-6, STO-13-6 and STO-15-6 sites, which had higher concentrations for Cu and Zn (Table 3). The concentration levels of these soil samples fall under Criterion C, which presents some usage constraints (e.g., residential construction, parks and green spaces).

For comparative purposes, the geochemical background data from the databases of the Ministère des Ressources naturelles et de la Faune [34], which provides the concentration of various metals obtained from the soil and sediment samples (natural background) for sector A4 of the Appalachian geological region, were examined. Sector A4 covers a large part of the Appalachian region and encompasses our study area. Based on the databases [34], the values obtained for average concentration of As, Cu, Pb and Zn in the soil and sediments are 11.1 mg kg^−1^ (SD 10.1), 11.5 mg kg^−1^ (SD 66.5), 15.3 mg kg^−1^ (SD 17.5) and 67.3 mg kg^−1^ (SD 82.3), respectively. If these values are compared to those obtained in the soil samples (Table 3), the concentration exceeds the background rates by factors of 1.5 to 20 for some sites (e.g., STO-10-6, STO-13-6, STO-15-6), and is two to three times higher for many other soil samples (e.g., STE-8-6, STE-10-6, MAS-13-3), excluding arsenic. Records show relatively high concentrations of Cu (120–130 mg kg^−1^) and Pb and Zn (55–65 and 440–1,500 mg kg^−1^) at the STO-13-6, STO-10-6 and STO-15-6 sites (Table 3). The high concentration of Cu and Zn at some of the sites probably originates from former mining sites and may even have been carried downstream to the Richmond area [24]. It is also possible that the metal content in the soil samples from the Richmond area comes from airborne dust produced by industrial activities some 40–60 kilometers away (Bécancour industrial site). It is also possible that contamination detected in surface soils (0–20 and 20–40 cm) comes from more remote atmospheric sources. At present, no regional study was conducted to evaluate the quality of the surface soils of this area.

### 4.3. Level of Contamination of Agricultural Area Soils

Table 4 shows the total concentrations of heavy metals obtained for subsurface horizons (0–20 cm) in the agricultural and riparian areas. In an attempt to assess the potentially polluting atmospheric fallout from nearby industrial sites, only the surface horizon results are presented (Table 4). A comparison of the soils from the agricultural sites (WEN-1, CLO-1, SNI-1) against those from the riparian sites (RIC-1, RIC-2, RIC-9, WIN-2, WIN-3, MAS-1, MAS-6) shows generally lower concentrations for agricultural area soils. The maximum values obtained for heavy metals such as Cu, Ni and Zn are 18, 11 and 24 mg kg^−1^ for agricultural sites and 47, 42 and 95 mg kg^−1^ for riparian soils, respectively. Based on the MDDEP’s soil contamination criteria [18], none of the soil samples taken from the farm woodlots could be qualified as contaminated. However, Cu, Pb and Zn concentrations exceed natural background levels [35]; these values are about twice as high as the background levels (Table 4).

In addition, Al levels are slightly higher in the agricultural area soils than in the riparian soils. The maximum value detected in the agricultural soils is 12,000 mg kg^−1^, compared to 7,100 mg kg^−1^ in the riparian soils. The higher concentrations of Al in the agricultural soils may be associated with the inflow of Al dust from local manufacturing plants, including one aluminum smelter and an aluminum products recycling plant found at the Bécancour industrial site. Easterly and northeasterly winds may have carried dust and pollutants to neighbouring farmland (Figure 1).

Highly variable heavy metal concentrations have been found in surface soils (Ap horizon) for different regional agricultural soils in Québec [36] with minimum and maximum Al values ranging from 4,820 to 49,010 mg kg^−1^, with a mean value of 20,370 mg kg^−1^. Clay matrix soils generally had higher Al concentrations due to the composition of the (octahedral) clay sheets, which often consist of aluminum hydroxide [37]. For sandy soils in the Québec study, the mean Al concentrations were 10,000 mg kg^−1^ [36], similar to those measured in the agricultural areas (control sites) in the present study, which are also made up of a sand-dominated matrix [34]. In fact, this mean value of 10,000 mg kg^−1^ (Al) could represent background concentrations. On the other hand, Pb concentrations in soils in the agricultural areas range from 12 to 22 mg kg^−1^, which is slightly greater than the values obtained for natural Pb concentrations in the region’s soils and sediments [34]. The cultivated soils in agricultural areas are also probably contaminated by aluminum or other heavy metals to various degrees.

A recent study conducted by the Ministry of Environment [38] confirms an increase in heavy metals in Quebec farmland, including Pb and Cd in intensive agricultural zones. Another study realized on agricultural soils in southern Québec [39] also shows that the spreading of fertilizer and waste (e.g., liquid swine manure) may cause soil contamination by heavy metals over the long term, particularly copper and zinc. It is estimated that the concentration of copper and zinc in soil fertilized with swine manure over a period of 25 years can be 2–3 times higher (21.0 and 84 mg kg^−1^ for Cu and Zn) than the levels measured in unamended soils [39]. Note that the critical thresholds for Cu and Zn levels in agricultural soils is roughly 9 and 14 mg kg^−1^, respectively [40]. Given that these values are recorded in surrounding cultivated land, the issue of the quality of agricultural products (crops and forage) fed to livestock or destined for human consumption must be further addressed in detail. In a context where there is a risk of increased use of animal excrement and other chemical fertilizers to improve farmland [37], a substantial increase in heavy metals in these soils and in the agricultural food products grown in them is to be expected. The effect of a potential increase in heavy metals in agricultural food products and their impact on human health and the food webs will therefore have to be better assessed. However, efforts have been made over the past few years to attempt to reduce the impact of fertilizing waste on soil by adding probiotic products to animal feed and through better feed apportioning [39].

## 4. Conclusions

This comparative analysis shows that soils in riparian areas are affected to a greater extent by heavy metal contamination than the samples taken from the control sites (woodlots) in agricultural areas. The highest levels measured in riparian soils are 10 to 20 times greater than those in the region’s soils and sediments (background levels) [35], *i.e.*, 120 to 130 mg kg^−1^ for Cu and 440 to 1,500 mg kg^−1^ for Zn. Pb concentrations also show values that are above background levels but are less pronounced. For agricultural area soils, the heavy metal concentrations are lower than in the riparian soils, though Cu, Ni and Pb concentrations exceed soil and sediment background levels [35]. This heavy metal contamination of agricultural area soils may possibly be associated with atmospheric fallout from industrial sites found some 30–60 kilometers from the sampling sites, while the contamination of riparian soils basically stems from the pollutants transported by the Saint-François and Massawippi Rivers, though part of the contamination may also originate from the inflow of surrounding pollutants. The next stages of the study will consist in increasing the number of control sites in agricultural and riparian areas in order to obtain better spatial representation of soil contamination on a regional scale and thus better assess the potential impact of the contamination on the receiving environment. At the same time, isotopic Pb measurements are being considered to determine the origin and distinguish the different sources of pollution of the agricultural and riparian areas and thus supplement the data for research currently under way [16].

## Figures and Tables

**Table 1.** Means, medians, standard deviations and ranges for the properties of the soil samples.

	Mean	Median	SD	Range
(n = 74) pH CaCl_2_	5.15	5.31	0.90	3.45–7.09
Organic C (% dry soil)	0.67	0.66	0.37	0.05–1.56
Texture analysis (SCWG) (n = 27)				
Diameter (mm)				
Sand (2.0–0.10) (%)	61.8	63.6	14.1	35.6–94.3
Silt (0.10–0.05) (%)	28.2	27.2	12.6	3.3–52.1
Clay (≤0.002 (%)	10.1	10.3	11.2	2.4–25.1

Eolian deposits (≥90% of sand)				

**Table 2.** General geochemical characteristics of the soil samples collected in some sectors (RIC, STE, WIN, MAS, WEN, CLO). The total concentrations of various metals were determined with inductively coupled plasma mass spectrometry (ICP-MS).

Unit (mg kg^−1^)	Max. values	Min. values	Average	Median	25th percentile	75th percentile
As	**38**	1.1	10.0	8.59	6.21	12.32
Al*	12,000	3 600	6 640	6,250	5,300	7 025
Cd	5	0.03	0.66	0.48	0.33	0.72
Cr	37	12	22.1	23.0	18.75	26.0
Cu	**120**	5	39.9	31.0	28.0	43.0
Ni	44	26	31.6	34.5	29.75	37.5
Pb	**149**	3.2	48.0	21.9	16.75	34.46
Zn	**1,500**	10	146.8	93.0	65.0	111.0

The values of Al were calculated on 12 soil samples. The values in bold exceed level A of the MDDEP’s generic criteria of contaminated soils [18].

**Table 3.** Concentration of heavy metals for different soil samples collected in riparian areas (Ulverton, Richmond, Windsor and Massawippi areas). The total concentrations of the various metals were determined with inductively coupled plasma mass spectrometry (ICP-MS).

Unit (mg kg ^−1^) Area/Site	As	Al	Cd	Cr	Cu	Ni	Pb	Zn
Richmond (RIC)								
Ulverton (ULV)								
STO-1-3	1	3,600	0.03	12	5	33	3	10
STO-10-6	16	-	2.9	37	95	36	55	440
RIC-2-8	ND	6,800	ND	19	25	22	18	37
RIC-3-8	ND	5,000	ND	23	46	42	22	85
RIC-9-8	ND	7,100	ND	27	47	39	22	95
STE-8-6	12	6,200	1	30	43	37	22	130
STO-13-6	15	-	0.71	23	120	30	30	88
STO-15-6	19	-	4.9	30	130	44	65	1,500

Windsor (WIN)								
WIN-2-8	ND	5,400	ND	19	29	30	13	49
WIN-3-8	ND	7,000	ND	23	35	36	15	65
STE-1-3	1.6	4,500	0.21	17	26	26	12	60
STE-10-6	6.0	-	0.59	17	30	30	10	80

Massawippi (MAS)								
MAS-13-3	4.5	6,200	0.19	20	63	31	16	93
MAS-13-8	ND	6,300	ND	22	40	29	17	64

A level*	6	-	1.5	85	40	50	50	110
B level	30	-	5	100	100	100	500	500
C level	50	-	20	800	500	500	1,000	1,500

* Generic criteria (A, B and C levels) used for classification of contaminated soils [18]; ND : Not Detected.

**Table 4.** Concentration of heavy metals in riparian and agricultural zones. The data represent the values obtained for soil samples (*n* = 18) collected in the subsurface horizons (0–20 cm).

	Riparian zone (RIC-1, RIC-2, RIC-9,WIN-2, WIN-3, MAS-1, MAS-6)			Agricultural zone (Farm woodlots) (WEN-1, CLO-1, SNI-1)		

	Mean	Max.	Min.	Mean	Max.	Min.
Unit (mg kg^−1^)						
As	8.82	9.16	7.20	ND	ND	ND
Al	6,266	7,100	5,000	10,800	12,000	9,600
Cd	0.48	0.60	0.32	ND	ND	ND
Cu	37	47	25	12.5	18	7
Cr	22.2	27	19	6.5	10	3
Ni	32.2	42	22	7	11	3
Pb	19.97	25.25	13	17	22	12
Zn	69.8	95	24	19.5	24	15

ND: Not Detected						
